# Chemical Modifications of PhTX-I Myotoxin from *Porthidium hyoprora* Snake Venom: Effects on Structural, Enzymatic, and Pharmacological Properties

**DOI:** 10.1155/2013/103494

**Published:** 2012-12-19

**Authors:** Salomón Huancahuire-Vega, Daniel H. A. Corrêa, Luciana M. Hollanda, Marcelo Lancellotti, Carlos H. I. Ramos, Luis Alberto Ponce-Soto, Sergio Marangoni

**Affiliations:** ^1^Department of Biochemistry, Institute of Biology, State University of Campinas (UNICAMP), P.O. Box 6109, 13083-970 Campinas, SP, Brazil; ^2^Department of Organic Chemistry, Institute of Chemistry, University of Campinas (UNICAMP), Campinas, SP, Brazil; ^3^Biotechnology Laboratory (LABIOTEC), Department of Biochemistry, Institute of Biology, University of Campinas (UNICAMP), Campinas, SP, Brazil; ^4^National Institute of Science and Technology for Structural Biology and Bioimaging, Rio de Janeiro, RJ, Brazil

## Abstract

We recently described the isolation of a basic PLA_2_ (PhTX-I) from *Porthidium hyoprora* snake venom. This toxin exhibits high catalytic activity, induces *in vivo* myotoxicity, moderates footpad edema, and causes *in vitro* neuromuscular blockade. Here, we describe the chemical modifications of specific amino acid residues (His, Tyr, Lys, and Trp), performed in PhTX-I, to study their effects on the structural, enzymatic, and pharmacological properties of this myotoxin. After chemical treatment, a single His, 4 Tyr, 7 Lys, and one Trp residues were modified. The secondary structure of the protein remained unchanged as measured by circular dichroism; however other results indicated the critical role played by Lys and Tyr residues in myotoxic, neurotoxic activities and mainly in the cytotoxicity displayed by PhTX-I. His residue and therefore catalytic activity of PhTX-I are relevant for edematogenic, neurotoxic, and myotoxic effects, but not for its cytotoxic activity. This dissociation observed between enzymatic activity and some pharmacological effects suggests that other molecular regions distinct from the catalytic site may also play a role in the toxic activities exerted by this myotoxin. Our observations supported the hypothesis that both the catalytic sites as the hypothetical pharmacological sites are relevant to the pharmacological profile of PhTX-I.

## 1. Introduction

Phospholipase A_2_ (PLA_2_; EC 3.1.1.4) enzymes catalyse the hydrolysis of acyl ester bond of 1,2-diacyl-3-*sn*-phosphoglycerides at the *sn*-2 position, with a requirement for a Ca^2+^ [1]. PLA_2_ enzymes from snake venom are quite fascinating from both biological and structural point of view. Despite their structure being conserved, they exhibit a wide range of pharmacological activities, including neurotoxicity [2], myotoxicity [3, 4], and cardiotoxicity [5] as well as anticoagulant, hemolytic [6], antiplatelet [7], hypotensive [8], hemorrhagic [9], and edema inducing effects [10]. However, not all the PLA_2_ enzymes induce all these pharmacological effects. In general, an individual PLA_2_ exhibits one or more specific pharmacological effects [11, 12].

Within the family Viperidae, two distinct types of venom PLA_2_ molecules have been described, all of them sharing a high degree of homology both in primary and three-dimensional structure [13]: the “classical” PLA_2_, that present an invariant Asp49 residue that plays a key role in catalysis; and the “PLA_2_ homologues,” devoid of enzymatic activity, that present the substitution of Asp49 by Lys49 or, less frequently, by Ser, Arg, Gln, or Asn [14, 15]. Despite their difference in catalysis, both the Asp49 and the Lys49 proteins are able to induce various pharmacological effects [16]. Thus the structure-function relationship among this group of proteins is subtle and complicated [1]. In some cases, the pharmacological effects result from their enzymatic activities, probably through the action of the products of hydrolysis, lysophospholipids, and fatty acids, that alter cell membrane shape and permeability [17, 18] but for many of them, the pharmacological effects are independent of their enzymatic activities, such as Lys49 PLA_2_ myotoxins, which lack hydrolytic activity and therefore act via another mechanism, which is only partially understood. A site close to the C-terminus, comprising a variable combination of basic and hydrophobic amino acids, has been identified as being responsible for toxicity [19].

In a previous work, we showed that* Porthidium hyoprora *snake venom is a rich source of PLA_2_ enzymes. Additionally, we purified a myotoxic PLA_2_ (PhTX-I) to homogeneity in reverse-phase HPLC, which constitutes of a single polypeptidic chain, has a molecular mass of 14.249 Da, and whose amino acid sequence exhibits high identity with other myotoxic Asp49 PLA_2_ [20]. We also demonstrated that PhTX-I (20 *μ*g/mL) caused edema, *in vivo *creatine kinase release, C2C12 skeletal muscle myoblasts cytotoxicity, and neuromuscular blockade of chick biventer cervicis muscle preparations. However, it is still unknown whether these pharmacological effects were mediated by the phospholipase catalytic activity of PhTX-I or not. One of the strategies employed for the elucidation of the relationship between catalytic activity and pharmacological effects of PLA_2_ is based on the chemical modification of specific residues in these enzymes [21]. Using this approach, a dissociation of pharmacological effects and enzymatic activity for various PLA_2_ has been observed, suggesting the presence of separate enzymatic and pharmacological active site(s) contained within their amino acid sequences [22, 23]. In the present study, we investigated the effects of chemical modifications of specific amino acid residues (His, Tyr, Lys, and Trp), performed in PhTX-I, on their enzymatic, structural, and pharmacological properties.

## 2. Material and Methods

### 2.1. Reagents

2,4′-Dibromoacetophenone (BPB), 4-nitrobenzenesulfonyl fluoride (NBSF), 2-nitrobenzenesulfonyl chloride (NPSC), 4-nitro-3-(octanoyloxy) benzoic acid, and other reagents were from Sigma Chemical Co. (St. Louis, MO, USA); acetic anhydride (AA) was from Merck.

### 2.2. Purification of PhTX-I

The PhTX-I PLA_2_ from *Porthidium hyoprora *venom was purified by reverse phase HPLC [20]. Briefly, 5 mg of whole venom was dissolved in 200 *μ*L of buffer A (0.1% TFA) and centrifuged at 4500 g; the supernatant was then applied to a *μ*-Bondapak C18 column (0.78 × 30 cm; Waters 991-PDA system), previously equilibrated with buffer A for 15 min. The elution of the protein was then conducted using a linear gradient (0%–100%, v/v) of buffer B (66.5% Acetonitrile in buffer A) at a constant flow rate of 1.0 mL/min. The chromatographic run was monitored at 280 nm of absorbance, and after elution the fraction was lyophilized and stored at −40°C.

### 2.3. Chemical Modifications

Modification of His residues with 2,4′-Dibromoacetophenone (BPB) was carried out as previously described [21]. Briefly, 3 mg of PhTX-I were dissolved in 1 mL of 0.1 M Tris-HCl containing 0.7 mM EDTA (pH 8.0) and 150 *μ*L of BPB (1.5 mg/mL, in ethanol), and the mixture incubated for 24 h at 25°C. Modification of Lys residues with acetic anhydride (AA) was performed at a protein : reagent molar ratio of 1 : 50 [21]. PhTX-I (3 mg) was dissolved in 1.5 mL of 0.2 M Tris-HCl buffer at pH 8.0, and 10 *μ*L of AA was added and the mixture was incubated for 1 h at 25°C. Tyr residues were modified by treatment with 4-nitrobenzenesulphonyl fluoride (NBSF) as previously described [24]. Briefly, 1 *μ*mol of PhTX-I (10 *μ*mol of Tyr) was dissolved in 14 mL of 0.1 M Tris-HCl (pH 8.0) and incubated with 10 *μ*mol, of NBSF for 20 h at 25°C. Modification of Trp residues was performed according to Takasaki et al. [25]. Briefly, 9 mg of PhTX-I were dissolved in 4 mL 50% acetic acid containing 1 mg of 2-nitrobenzenesulfonyl chloride (NPSC) and incubated for 1 h at 25°C. In all cases, excess reagent was removed by ultrafiltration through a Millipore's Amicon Ultra-15 membrane and washed with distilled water, followed by lyophilization.

### 2.4. Amino Acid Analysis

Amino acid analysis was performed on a Pico-Tag Analyzer (Waters Systems) as described by Heinrikson and Meredith [26]. Native PhTX-I PLA_2_ and their modified derived samples (30 *μ*g) were hydrolyzed at 105°C for 24 h, in 6 M HCl (Pierce sequencing grade) containing 1% phenol (w/v). The hydrolysates were reacted with 20 *μ*L of derivatization solution (ethanol : triethylamine : water : phenylisothiocyanate, 7 : 1 : 1 : 1, v/v) for 1 h at room temperature, after which the PTC amino acids were identified and quantified by HPLC, by comparing their retention times and peak areas with those from a standard amino acid mixture.

### 2.5. Electrophoresis

Native PhTX-I PLA_2_ and their modified derivatives were examined by Tricine SDS-PAGE in a discontinuous gel and buffer system, under reducing and nonreducing conditions [27]. The molecular mass markers used were (in kDa): phosphorylase B—97.0, albumin—66.0, ovalbumin—45.0, carbonic anhydrase—30.0, soybean trypsin inhibitor—20.1, and *α*-lactalbumin—14.4.

### 2.6. Mass Spectrometry

An aliquot (4.5 *μ*L) of the modified proteins was injected in C18 (100 *μ*m × 100 mm) RP-UPLC (nanoAcquity UPLC, Waters) coupled with nanoelectrospray tandem mass spectrometry on a Q-Tof Ultima API mass spectrometer (MicroMass/Waters) at a flow rate of 600 nL/min. The gradient was 0%–50% acetonitrile in 0.1% formic acid over 45 min. The instrument was operated in MS continuum mode, and the data acquisition was from *m/z* 100–3.000 at a scan rate of 1 s and an interscan delay of 0.1 s. The spectra were accumulated over about 300 scans and the multiple charged data by the mass spectrometer on the *m/z* scale were converted to the mass (molecular weight) scale using maximum entropy-based software supplied with Masslynx 4.1 software package. The processing parameters were output mass range 6.000–20.000 Da at a “resolution” of 0.1 Da/channel; the simulated isotope pattern model was used with the spectrum blur width parameter set to 0.2 Da; the minimum intensity ratios between successive peaks were 20% (left and right). The deconvoluted spectrum was then smoothed (2 × 3 channels, Savitzky Golay smooth) and the mass centroid values obtained using 80% of the peak top and a minimum peak width at half height of 4 channels.

### 2.7. Circular Dichroism

Circular dichroism (CD) spectra of native PhTX-I PLA_2_ and their modified derivatives were recorded with a JASCO model J-720-ORD 306 spectropolarimeter equipped with a thermoelectric sample temperature controller (Peltier system) following standard procedures previously described [28]. After centrifugation at 4000 g for 5 min, samples (1–4 *μ*M protein in 10 mM sodium phosphate, pH 8) were transferred to a 10-mm path-length quartz cuvette. Circular dichroism spectra in the wavelength range 260 to 200 nm were collected, using a bandwidth of 1 nm and a response time of 1 s. Data collection was performed at 25°C with 50 nm/min scanning speed. At least ten scans were accumulated for each sample, and all spectra were corrected by subtraction of buffer blanks. The estimation of secondary structure elements was performed using the CDNN Deconvolution software (version 2.1), and Origin 7.5 (OriginLab) was used for graphics and analysis.

### 2.8. PLA_2_ Activity

PLA_2_ activity was measured using the assay described by Cho and Kezdy [29] and Holzer and Mackessy [30] modified for 96-well plates. The standard assay mixture contained 200 *μ*L of buffer (10 mM Tris-HCl, 10 mM CaCl_2_, and 100 mM NaCl, pH 8.0), 20 *μ*L of substrate 4-nitro-3-(octanoyloxy) benzoic acid (3 mM), 20 *μ*L of water, and 20 *μ*L of PhTX-I PLA_2_ or their modified derivatives (1 mg/mL) in a final volume of 260 *μ*L. After adding proteins (20 *μ*g) the mixture was incubated for up to 40 min at 37°C, measuring absorbance at intervals of 10 min. The enzyme activity, expressed as the initial velocity of the reaction (*V*_*o*_), was calculated based on the increase of absorbance after 20 min. All assays were done in triplicate, and the absorbances at 425 nm were measured with a VersaMax 190 multiwell plate reader (Molecular Devices, Sunnyvale, CA).

### 2.9. Inhibition

The inhibitory effects of EDTA or low molecular weight heparin from porcine intestinal (Mr 6.000 Da) on pharmacological and enzymatic activities of PhTX-I were assessed by incubating the enzyme with 1 mM solution of this chelating agent or a heparin : toxin molar ratio of 2 : 1 for 30 min at 37°C. The inhibition of PLA_2_ activity of PhTX-I by crotapotins F2 and F3 from *Crotalus durissus collilinetaus *also was evaluated by incubating the two proteins (1 : 1, w/w) for 30 min at 37°C and then assaying the residual enzyme activity.

### 2.10. Chick Biventer Cervicis Muscle Preparation (BCP)

Animals were anesthetized with halothane and sacrificed by exsanguination. The biventer cervicis muscles were removed and mounted under a tension of 0.5 g, in a 5 mL organ bath (automatic organ multiple-bath LE01 Letica Scientific Instruments. Barcelona, Spain) at 37°C containing aerated (95% O_2_-5% CO_2_) Krebs solution (pH 7.5) of the following composition (mM): NaCl 118.70, KCl 4.70, CaCl_2_ 1.88, KH_2_PO_4_ 1.17, MgSO_4_ 1.17, NaHCO_3_ 25.00, and glucose 11.65. Contracture to exogenously applied acetylcholine (ACh; 110 *μ*M for 60 s) and KCl (20 mM for 130 s) was obtained in the absence of field stimulation, before and after the addition of a single dose of PhTX-I PLA_2_ (1.4 *μ*M) or their modified derivatives (1.4 *μ*M). A bipolar platinum ring electrode was placed around the tendon, which runs the nerve trunk supplying the muscle. Indirect stimulation was performed with a (MAIN BOX LE 12404 Panlab s.l. Powerlab AD Instruments Barcelona, Spain) stimulator (0.1 Hz, 0.2 ms, 3-4 V). Muscle contractions and contractures were isometrically recorded by force-displacement transducers (Model MLT0201 Force transducer 5 mg–25 g Panlab s.l. AD Instruments Pty Ltd. Spain) connected to a PowerLab/4SP (OUAD Bridge AD Instruments, Barcelona, Spain).

### 2.11. Myotoxic Activity

Groups of five Swiss mice (18–20 g) received an intramuscular injection (i.m.) of 20 *μ*g of PhTX-I PLA_2_ or their modified derivatives dissolved in 100 *μ*L of PBS, in the gastrocnemius. A control group received 100 *μ*L of PBS (0.12 M NaCl, 0.04 M sodium phosphate, pH 7.2). Three hours after injection, blood was collected from the tail into heparinized capillary tubes, and the plasma creatine kinase (CK; EC 2.7.3.2) activity was determined by a kinetic assay (Sigma 47-UV). Activity was expressed in U/L, one unit defined as the phosphorylation of 1 *μ*mol of creatine/min at 25°C.

### 2.12. Edema-Forming Activity

The ability of PhTX-I PLA_2_ and their modified derivatives to induce edema was studied in groups of five Swiss mice (18–20 g). Fifty *μ*L of phosphate-buffered saline (PBS; 0.12 M NaCl, 0.04 M sodium phosphate, pH 7.2) with toxins (1 *μ*g/paw) were injected in the subplantar region of the right footpad. The left footpad received 50 *μ*L of PBS, as a control. The paw volume was evaluated plethysmographically (Model 7140 Plethysmometer, Ugo Basile, USA), immediately before the injection (basal) and after 1 h. Edema-forming activity was expressed as the percentage of the increase in volume of the right foot pad in comparison to the left foot pad (control). The equation for calculation of the percentage of edema in toxins injected paw was

(1) $$\begin{array}{c} \%\text{edema}=\left[\left(\frac{T_{x}\times 100}{T_{0}}\right)-100\right], \end{array}$$

where *T*_*x*_ is the edema (volume) measured at each time interval and *T*_0_ is the volume of the paw (intact, zero time before toxins injection). The percentage of edema calculated was subtracted from the matched values at each time point in the saline injected hind paw (control).

### 2.13. Cytotoxic Activity

#### 2.13.1. Maintenance of NG97 and NCIH-3T3 Cell Culture

Cytotoxic activity was assayed on NG97 and NCIH-3T3 cells, which were grown in plastic flasks (25 cm^2^) with RPMI 1640 medium (Cultilab, Campinas, SP, Brazil), supplemented with 2% L-glutamine, 120 *μ*g/mL garamycin, and 13% inactivated fetal bovine serum (complete medium). The cultures were incubated at 37°C in an atmosphere containing 5% of CO_2_. Medium was changed every 48 h, and when the culture reached confluence, the subculture was performed by treatment with trypsin and versene (Adolfo Lutz, São Paulo, SP, Brazil). Variable amounts of native PhTX-I and chemically modified derivatives were diluted in assay medium and added to cells in 96-well plates. Experiments were carried out in triplicate.

#### 2.13.2. Cellular Viability by Neutral Red Uptake Assay

This assay was done according to the method described by Borenfreund and Borrero [31]. After a 4 h incubation with serum-free medium containing 50 *μ*g of neutral red/mL the cells were washed quickly with PBS and then 0.1 mL of a solution of 1% (v/v) acetic acid : 50% (v/v) ethanol was added to each well to extract the dye. After shaking for 10 minutes on a microtitre plate shaker, the absorbance was read at 540 nm. The cell death was determined in comparison of the absorbance obtained from nontreated cells.

### 2.14. Statistical Analyses

Results were reported as mean ± SEM. The significance of differences among means was assessed by analysis of variance followed by Dunnett's test, when several experimental groups were compared with the control group. Differences were considered statistically significant if *P* < 0.05.

## 3. Results

**Figure 1:**
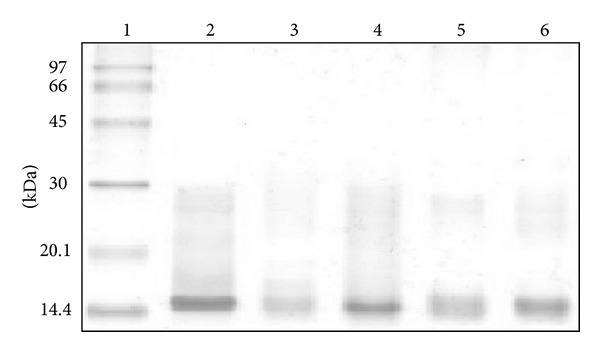
Electrophoretic profile in Tricine SDS-PAGE of native PhTX-I and their chemically modified derivatives. (1) Molecular mass markers, (2) native PhTX-I, (3) AA-treated PhTX-I, (4) NPSC-treated PhTX-I, (5) NBSF-treated PhTX-I, and (6) BPB-treated PhTX-I.

Table 1 shows the result of analysis of amino acid composition of the modified proteins of PhTX-I PLA_2_ compared to the amino acid sequence of native PhTX-I. After chemical treatment a single His, 4 Tyr, and 7 Lys residues were modified by BPB, NBSF, and AA, respectively. With the methodology employed it was not possible to determine the changes in Trp residues. In all these cases the exact position of the groups modified is unknown. The homogeneity of the modified proteins was evaluated by SDS-PAGE, as shown in Figure 1. Both native PhTX-I and modified forms migrated similarly by electrophoresis, indicating the presence of a single band electrophoretic for each protein, demonstrating the high homogeneity of the samples as well as no detectable change in the apparent molecular weight of the modified forms of the PhTX-I.

**Figure 2:**
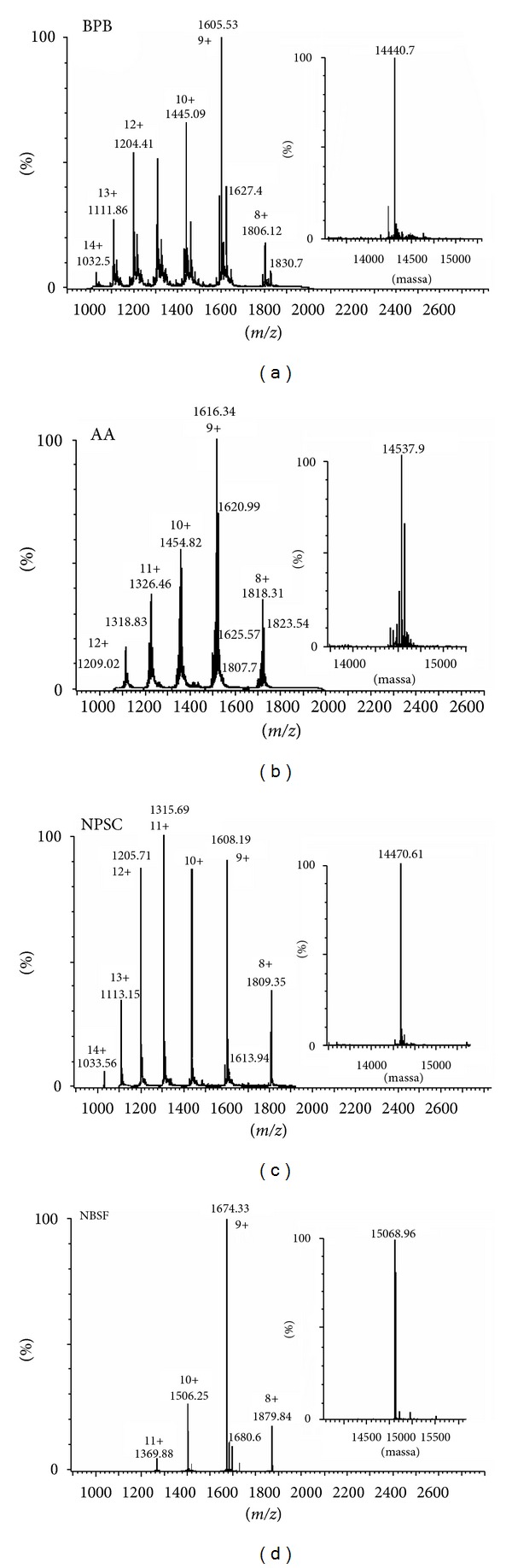
Mass determination by ESI mass spectrometry of the chemically modified derivatives of PhTX-I. Raw and deconvoluted mass spectra of BPB-treated PhTX-I (a), AA-treated PhTX-I (b), NPSC-treated PhTX-I (c), and NBSF-treated PhTX-I (d).

Molecular mass values of the modified proteins were determined by ESI mass spectrometry. Figures 2(a), 2(b), 2(c), and 2(d) show the mass spectrum of modified forms of PhTX-I, by BPB, AA, NPSC, and NBSF, respectively; each peak represents the protein carrying a different number of charges (protons). Figures inserted in each spectrum show the deconvolution of the spectra obtained of the modified proteins. The modified protein in His, Lys, Trp, and Tyr residues had molecular mass of 14440.7, 14537.9, 14470.1, and 15068.9 Da, respectively.

**Figure 3:**
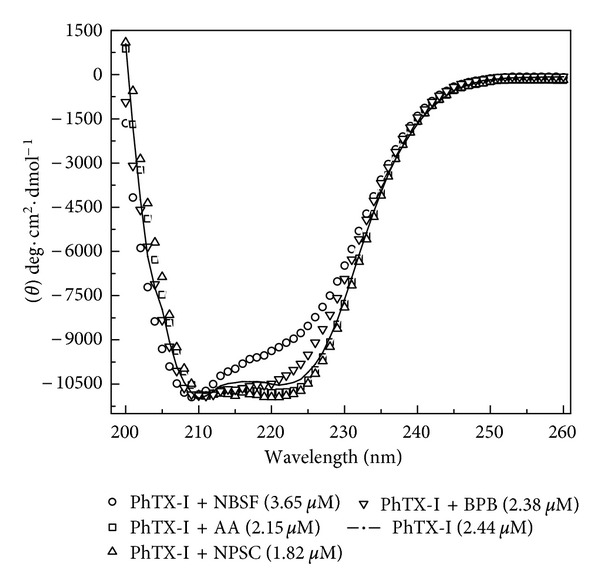
Far-UV circular dichroism spectra of native PhTX-I and their chemically modified derivatives. Native PhTX-I (continuous lines) and their chemically modified derivatives (symbols): NBSF-treated PhTX-I (circles), BPB-treated PhTX-I (inverted triangles), NPSC-treated PhTX-I (open triangles), and AA-treated PhTX-I (squares).

To determine whether the chemical modifications in PhTX-I caused changes in protein secondary structure, far-UV circular dichroism (CD) was used.  Figure 3 shows CD spectra of native PhTX-I and their modified forms which demonstrate two negative bands of similar magnitude (−11,000 to −10,500 deg·cm^2^·dmol^−1^) at 208 and 222 nm and a positive one at ~190 nm (data not shown), indicating a consistent content of *α*-helical structures. The exception was NBSF modified PhTX-I that lad lower signal at 222 nm than the unmodified protein. Based on the CDNN program analysis of the native PhTX-I spectrum, the contents of *α*-helices, *β*-sheets, and *β*-turns were 32%, 18%, and 17%, respectively. The contents of secondary structure of PhTX-I chemically modified by BPB, NPSC, or AA were very similar to native PhTX-I, with no significant changes in the CD spectrum between them, suggesting that secondary structure of protein remains practically unchanged. However, NBSF treatment resulted in a significant change (−10,500 to −9,000 deg·cm^2^·dmol^−1^) in molar ellipticity of the negative band at 222 nm, which by CDNN software analysis indicates an altered content of *α*-helices, *β*-sheets, and *β*-turns (30%, 19% and 18%, resp.).

**Figure 4:**
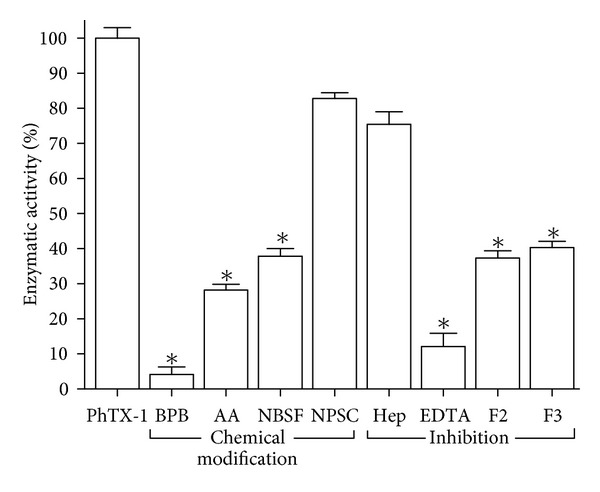
PLA_2_ activity of native PhTX-I PLA_2_ and their chemically modified derivatives with BPB, AA, NBSF, NPSC and inhibitory effect of heparin, EDTA, and crotapotins (F2 and F3) upon 4-nitro-3-(octanoyloxy) benzoic acid substrate *(*P* < 0.05).

The catalytic activity of native PhTX-I and their modifications were studied using the chromogenic substrate 4-nitro-3-(octanoyloxy) benzoic acid. The catalytic activity of native PhTX-I was almost completely abolished by BPB, but only partially reduced after modification of Tyr or Lys residues; NPSC did not cause a significant decrease in this activity (Figure 4). The incubation of native PhTX-I with crotapotins F2 and F3 from *C. d. collilinetaus* and EDTA diminished the activity; heparin did not significantly inhibit the catalytic activity (Figure 4).

**Figure 5:**
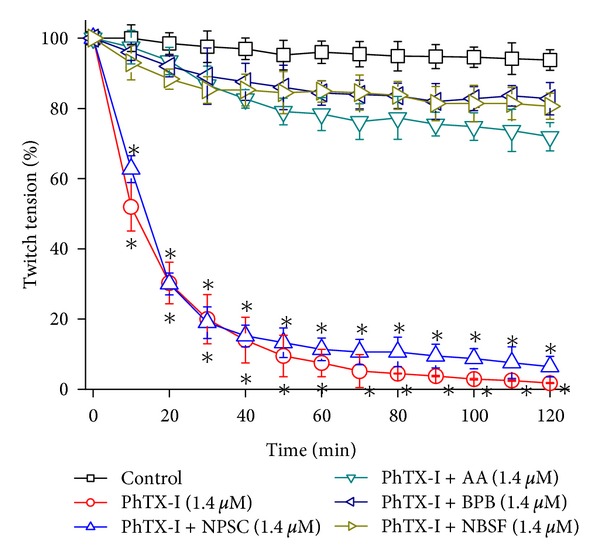
Neuromuscular blockade of chick biventer cervicis preparations incubated at 37°C with native PhTX-I and their chemically modified derivatives with BPB, AA, NBSF, and NPSC, all in concentration 1.4 *μ*M. The points are the mean ± SEM of six experiments. *(*P* < 0.05) compared to the twitch-tension before toxin addition.

Figure 5 shows the graphical representation of the blockade of the contractile response in the neuromuscular transmission (BCP) of native PhTX-I and modified derivatives. The native PhTX-I at a concentration of 1.4 *μ*M blocked the indirectly evoked contractions reaching 50% of the block in about 20 min. This activity was markedly diminished for all the PhTX-I chemically modified derivatives, except for modification of Trp where no significant change in force of contraction was produced by the modification.

**Figure 6:**
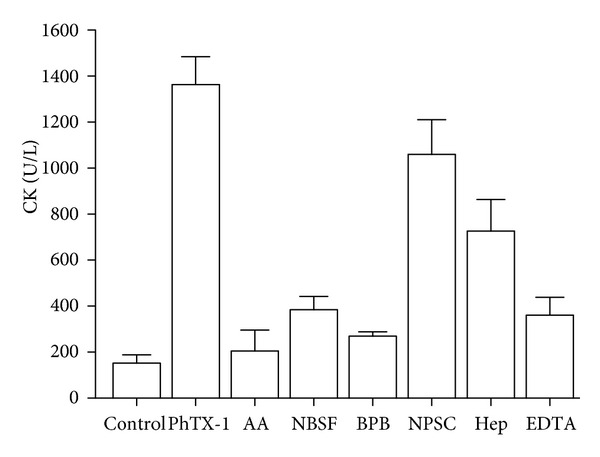
Increments in plasma CK activity after intramuscular injection of native PhTX-I their chemically modified derivatives with BPB, AA, NBSF, and NPSC (all 20 *μ*g/100 *μ*L) and incubation of PhTX-I (20 *μ*g) with heparin and EDTA. Controls were injected with 100 *μ*L of PBS. Three hours after injection, blood was collected, and serum levels were measured. Values are mean ± SEM of five mice at each time point.

Figure 6 shows the effect of the different chemical modifications on the myotoxic activities of PhTX-I, by time-course measurement of plasma levels CK after intramuscular injection of proteins. It can be seen that even when BPB and AA were the most effective reagents altering this activity, most of the treatments had some effect. Three hours after treatment started, the myotoxic effect was reduced by 85%, 80%, and 72% by treatment with AA, BPB, and NBSF, respectively. EDTA and heparin inhibited around 74% and 50% of this activity.

**Figure 7:**
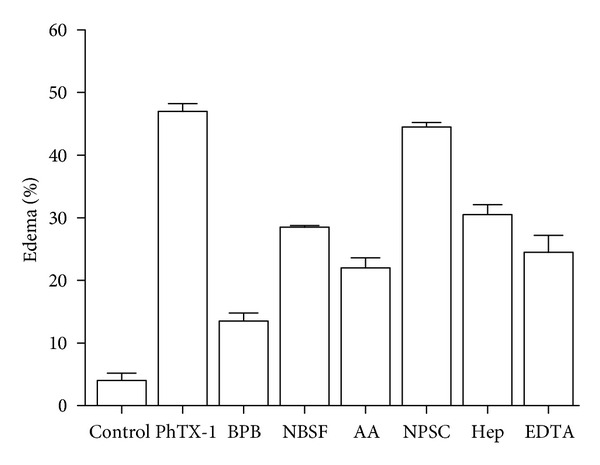
Edema-forming activity of native PhTX-I and their chemically modified derivatives with BPB, AA, NBSF, and NPSC (all 1 *μ*g) and incubation of PhTX-I (1 *μ*g/50 *μ*L) with heparin and EDTA. The toxins were injected s.c. in the footpad of mice. Edema was evaluated after 1 h injection and expressed as percent edema. Each point represents the mean ± SEM of five animals.

Regarding the edema-inducing effect, after alkylation with BPB, PhTX-I lost around 70% of its activity one hour after treatment, while modification of Lys and Tyr residues caused only a partial decrease of this activity (55% and 40%, resp.); however, NPSC did not inhibit this activity, as can be seen in Figure 7.

**Figure 8:**
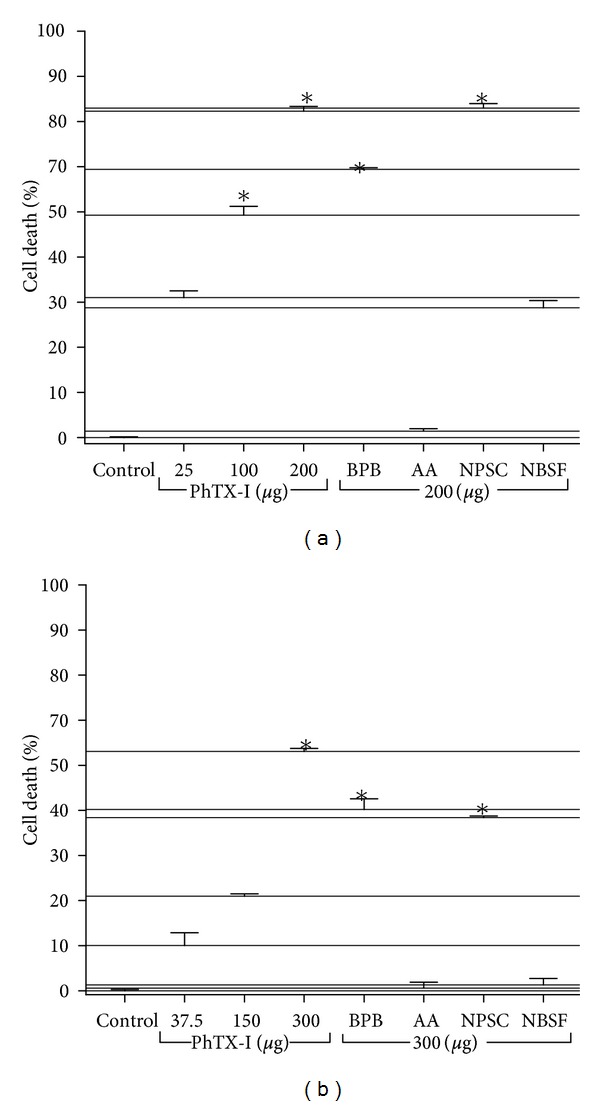
*In vitro* cytotoxic activity of native PhTX-I PLA_2_ and their chemically modified derivatives with BPB, AA, NBSF, and NPSC on NCIH-3T3 (a) and NG97 (b) cell culture. Cell lysis was estimated by neutral red uptake assay. The cell death was determined in comparison of absorbance obtained from nontreated cells. Experiments were carried out in triplicate. *(*P* < 0.05).

Native PhTX-I was cytotoxic to NIH-3T3 fibroblast cell line and to a lesser extent for the NG97 cell line derived from a human astrocytoma grade III (Figure 8). The cytotoxic activity of PhTX-I was independent of enzymatic activity, since BPB-treated PhTX-I was able to produce cytotoxicity in both cell types. After treatment with NPSC the cytotoxic activity was partially reduced. On the other hand, acetylation and sulfonylation of Lys and Tyr residues, respectively, reduced strongly the cytotoxic activity in both cell types.

## 4. Discussion

We recently described the isolation of a basic PLA_2_ (PhTX-I) from *P. hyoprora* using reverse phase HPLC [20]. This toxin exhibits high catalytic activity, shares various structural similarities with other “bothropics” PLA_2_, and has the conserved and essential Asp residue in position 49. PhTX-I induces *in vivo* myotoxicity, moderates footpad edema (at concentrations up to 10 *μ*g/mL and 0.5 *μ*g/mL, resp.), and causes *in vitro *neuromuscular blockade in chick biventer cervicis muscle preparations (at concentrations of 1.4 *μ*M). Here, we have described the chemical modifications of specific amino acid residues (His, Tyr, Lys, and Trp), performed in PhTX-I and how theses modifications affected the structural, enzymatic, and pharmacological properties of this myotoxin.

A first important observation was that after chemical treatment a single His, 4 Tyr, 7 Lys, and one Trp residues were modified by BPB, NBSF, AA, and NPSC, respectively (Table 1). These results were confirmed by mass spectrometry (Figure 2). The mass of native PhTX-I, 14249.22 Da [20], after treatment with NBSF (15068.96 Da) increased 819.74 Da (Figure 2(d)), demonstrating modification of fourth Tyr residues (up to 187.16 Da in each residue corresponding to reagent NBS that would be incorporated). Similarly, only three Tyr residues (7, 70, and 77) with the highest exposed surface areas in the notexin were modified by NBSF, suggesting that the seven remaining residues are “buried” within the molecule [32]. Acetylation of Lys residues of basic PLA_2_ myotoxins three (PrTX-I,-III and BnSP-7) caused a complete loss of basicity, being demonstrated by electrophoretic analysis [22, 33]; however AA-treated PhTX-I (14537.9 Da) (Figure 2(b)) shows that only seven Lys residues were modified, due to which there is an increase of 288.60 Da, equivalent to seven times the mass of acetyl radical (42 Da) incorporated into the amino group of K residues, showing similar behavior to the native protein in electrophoresis gel (Figure 1).

Similarly, an increase of 221.39 Da in NPSC-treated PhTX-I (Figure 2(c)) indicated a modification of a single Trp residue. By analogy with other results using PrTX-I and -III from *B. pirajai* [22], we would expect that, with the three Trp residues present in the structure of PhTX-I, this reagent should modify the residue with a larger area of exposure; it would be more easily attacked by the NPSC. Alkylation of His by BPB has been widely used to assess the role of enzymatic activity in the pharmacological actions of PLA_2_ [22, 23, 33–36]. PhTX alkylated with BPB had a molecular mass of 14440.7 Da (Figure 2(a)), which confirmed the modification of only one residue of His. His48 is a highly conserved amino acid residue in PLA_2_, which has a vital role in catalysis [37]. Since the enzymatic activity of the PhTX-I was almost completely abolished after this modification, His48 was likely the residue modified, because this amino acid is part of the catalytic triad of this protein family.

Examination of native PhTX-I by CD spectroscopy indicated that the predominant secondary structure of this PLA_2_ consisted of alpha-helices (Figure 3), in agreement with the results obtained for others as PLA_2_ from Taiwan cobra [38], PLA2A from *C. d. ruruima *[39], and BnIV from *B. newidii* [40]. The secondary structure of PhTX-I chemically modified derivatives did not alter significantly after modifications as evidenced by the CD spectra, which exhibited almost the same profile as that of native toxin. Only NBSF-treated toxin revealed detectable changes in secondary structure composition when compared to native toxin (Figure 3). This difference could be due to partial helix unfolding but the small change suggested that such unfolding was minimal. Kini [1] described that the alkylation by BPB does not affect the three-dimensional structure of PLA_2_ or its ability to bind phospholipids, but may alter the ability to interact with specific proteins or ligands. Thus, it is suggested that the chemical modifications performed in this study mainly affected the specific residues involved in such modifications and did not result in drastic conformational changes in the molecule.

The PhTX-I PLA_2_ is a Ca^2+^-dependent enzyme, with maximum activity at pH 8 and 40°C, reaching *V*_max⁡_and *K*_*m*_  of 11.76 nmoles/min and 1.96 mM, respectively[20]. Heparin slightly decreased enzymatic activity of PhTX-I, (25%) (Figure 4); similarly, this polyanionic compound resulted acting as negative allosteric modulator of PLA_2_ *C. d. cascavella* [41]. On the other hand, EDTA greatly decreased catalytic activity of PhTX-I (88%), as *β*-bungarotoxin and notexin, which were inhibited by EDTA even in the presence of an excess of Ca^2+^ [42]. The F2 and F3 crotapotins from *C. d. collilineatus *significantly inhibited the enzymatic activity of PhTX-I at approximately 55% (Figure 4), in agreement with BjIV PLA_2_ of *B. jararacussu*, which was inhibited by 50% in its catalytic activity by F7, F3, and F4 crotapotins from *C. d. terrificus*, *C. d. collilineatus,*  and *C. d. cascavella* [43]. These results suggest that crotapotins can bind to bothropics PLA_2_ a manner similar to that of crotalics PLA_2_, and raise the possibility that bothropic venoms may contain crotapotin-like proteins which inhibit the catalytic activity of PLA_2_.

Acetylation of Lys residues significantly reduced the enzymatic activity; however, a residual activity was detected, corresponding to 26% (Figure 4), similarly to MT-III (*B. asper*) and MT-I (*B. godmani*) [21]. The mode of specific acetylation is not clear, but there is some evidence of reduction of the calcium-binding capacity, thereby reducing the enzymatic activity of PLA_2_ [44]. In contrast, both myotoxic and cytotoxic effects were totally abolished, whereas a residual edematogenic effect remained (Figures 6, 7, and 8). These observations agree with previous studies in which the Lys of PrTX-I, PrTX-III, and BnSP-7 PLA_2_ were modified by acetylation [22, 33] and drastically decreased myotoxic, edema-inducing, and bactericidal activities. Acetylation of Lys residues in PhTX-I also decreased the blockade of the contractile response in the neuromuscular transmission, more than enzymatic activity (Figure 5). This greater effect on some pharmacological properties than on the enzymatic activity of Lys-modified PhTX-I demonstrates the dissociation between enzymatic and pharmacological activities and evidence of the existence of molecular regions, distinct from the catalytic site, which may be responsible for at least some of the pharmacological properties of these toxins, in agreement with previous studies [23, 34, 35].

Soares et al. [22] suggested that the bactericidal effect of PrTX-III and myotoxin III from *B. pirajai* and *B. asper*, respectively, might be related to overall basicity of the protein from the N-terminal helix and residues the 115–129 in the C-terminal region that is rich in aromatic and basic residues. This C-terminal region was identified as a structural determinant of this effect [45]. Short synthetic peptides representing the C-terminal region of PLA_2_ myotoxins showed cytolytic and muscle damaging activities similar to their parent proteins, although they display a lower potency [46–48]. Significant decrease of myotoxic activity after incubation with heparin confirms the involvement of C-terminal region of PhTX-I which is a basic myotoxin with high content of Lys residues [20].

His48 is a highly conserved residue in PLA_2_, which has an important role in catalysis [37]. Alkylation of His by BPB has been widely used to assess the role of enzymatic activity in the pharmacological actions of PLA_2_ [21, 35, 36, 49]. Here, alkylation of His at the active site of PhTX-I markedly abolished enzymatic activity (<4% residual activity) (Figure 4). Others have reported residual enzymatic activity following alkylation of His48, as *β*BuTX and notexin from *N. nigricollis* and *N. n. atra* PLA_2_, with values close to that found for BPB-PhTX-I (5%–8% of residual activity) [34]. Myotoxic, neurotoxic, and edema-forming activities of PhTX-I, were drastically reduced by this modification (Figures 5, 6, and 7); EDTA by treatment also affected myotoxicity and edematogenic activity (Figures 6 and 7), strengthening the hypothesis that phospholipid enzymatic hydrolysis is involved in these effects. Similarly, neurotoxic and myotoxic activities were inhibited almost completely after alkylation of His48 of PLA_2_ Basp-III (*B. asper*), PrTX-III (*B. pirajai*), BthTX-II (*B. jararacussu*), Cdc-9, and Cdc-10 (*C. d. cumanensis*) [21, 22, 35, 49], showing that these pharmacological effects are dependent on the catalytic activity. Since alkylation of the active site His48 completely abolished the catalytic activity and strongly attenuated these three pharmacological effects, Kini [1] suggested the hypothesis that PLA_2_ activity potentiates these pharmacological effects induced by *Bothrops* and *Crotalus *myotoxins.

In contrast, cytotoxic activity upon NG97 and NCIH-3T3 cells not was affected by His modification (Figure 8), suggesting that enzymatic activity is not required for this effects and that there are other molecular regions involved in cellular membrane perturbation. Our results agree with MTX-I and II PLA_2_ from *B. brazili*, which displayed cytotoxic activity against Jurkat lines independently of catalytic activity [50]. Some authors propose that cytotoxic activity on tumor cell lines is associated with apoptosis induction, considering the fact that PLA_2_ enzymes have been proposed to play a role in mediating apoptosis in various models, including cell lines [51]. The PLA_2_ activity is proposed to accelerate turnover of phospholipids, which may influence membrane changes that occur during apoptosis [52]. We suggested important role of Lys residues in cytotoxic effect, because PhTX-I treatment with AA abolished this activity.

Studies have been directed trying to understand the mechanisms involved in the inflammatory response induced by myotoxic PLA_2_ from several snake venoms [53–55]. However, the relationship between enzymatic activity and edema is contradictory [56]. It is assumed that myotoxic and edematogenic activities can be induced by different structural domains in these PLA_2_, or that a partial overlapping of these domains occurs [55, 57].

Residual enzymatic activity after sulfonylation of Tyr residues was 38% (Figure 4). Tyr52 and Tyr73 are part of the catalytic site of PLA_2_; giving structural support to stabilize the catalytic system [37], changes in this system would affect the enzymatic activity. Tyr-modified PhTX-I decreased myotoxic and neurotoxic activities more than the enzymatic activity (Figures 4, 5, and 6), once again indicating the dissociation between enzymatic and pharmacological activities. Cytotoxic activity of PhTX-I also was reduced drastically after modification by NBSF (Figure 8), indicating that Tyr residues would also be involved in this process. Zhao et al. [58] observed that myotoxic PLA_2_ have a set of Tyr residues located at the C-terminal region of the molecule. These Tyr may contribute to the hydrophobic-cationic combination proposed to play a role in myotoxicity and cytotoxicity [19, 46, 59]. PhTX-I show a Tyr residue in C-terminal region; alterations of this acid amino in this region of the molecule are causing reduction of toxicity of PhTX-I. However, the influence of conformational changes induced by NBSF in these effects cannot be discarded (Figure 3).

NPSC-treated PhTX-I did not significantly decrease enzymatic activity (Figure 4), suggesting that the modified Trp residue is not related to the catalytic system. In the same way, this modification showed no changes as compared to native PhTX-I, in edematogenic and cytotoxic activities (Figures 7 and 8); the myotoxic effect was minimally affected (Figure 6). In this sense Trp residues of PhTX-I have little or no direct action on the muscle. The Trp modifications of PhTX-I also maintained the action upon blockade of the contractile response in chick biventer cervicis muscle preparation (Figure 5). In contrast, modified Trp affected only the neurotoxic effect caused by MjTX PLA2-II [60], indicating the relevant role of this residue in this activity and suggesting that chemical modification could be interfering with the stability of the interaction between the monomers of this dimeric toxin, since Trp77 helps to maintain the homodimeric interaction. This suggests that the shift from dimeric to monomeric form of myotoxin may reduce the ability to affect the plasma membrane [60]. Because PhTX-I is a monomeric toxin, modified Trp would not affect the pharmacological activity of this toxin.

## 5. Conclusions

Our results indicate the critical role played by Lys and Tyr residues in myotoxic, neurotoxic activities and mainly in the cytotoxicity displayed by PhTX-I. His residue and, therefore, catalytic activity of PhTX-I are relevant for edematogenic, neurotoxic, and myotoxic effects, but not for its cytotoxic activity. Our observations supported the existence of pharmacological sites, distinct from the catalytic site, that contribute to the development of toxicity of these toxins and the hypothesis that the catalytic activity would potentiate the myotoxic and neurotoxic effects induced by snake venom PLA_2_. Finally, although a partial dissociation is shown, both the catalytic sites as the hypothetical pharmacological sites are relevant to the pharmacological profile of PhTX-I.

## Figures and Tables

**Table 1:** Amino acid composition of native PhTX-I and their chemically modified derivatives.

Amino acid	PhTX-I native^a^	PhTX-I modified^b^	PhTX-I modified^c^	PhTX-I modified^d^	PhTX-I modified^e^
Asp	11	10.92 (11)	10.9 (11)	10.72 (11)	10.56 (11)
Glu	6	5.98 (6)	6.12 (6)	5.92 (6)	6.37 (6)
Ser	3	3.37 (3)	3.15 (3)	3.21 (3)	3.03 (3)
Gly	11	11.43 (11)	11.25 (11)	11.39 (11)	11.13 (11)
His	2	1.74 (2)	1.94 (2)	1.6 (2)	0.65 (**1**)
Arg	7	7.62 (7)	6.56 (7)	7.15 (7)	7.42 (7)
Thr	6	6.34 (6)	6.09 (6)	6.35 (6)	6. 34 (6)
Ala	5	4.81 (5)	5.29 (5)	5.34 (5)	5.44 (5)
Pro	6	5.82 (6)	6.01 (6)	6.05 (6)	5.74 (6)
Tyr	10	10.24 (10)	9.79 (10)	5.94 (**6**)	10.21 (10)
Val	3	3.33 (3)	3.34 (3)	3.49 (3)	3.49 (3)
Met	1	1.47 (1)	1.17 (1)	1.18 (1)	1.45 (1)
Cys	14	13.96 (14)	13.98 (14)	13.74 (14)	13.69 (14)
Ile	2	2.47 (2)	2.35 (2)	2.27 (2)	2.22 (2)
Leu	8	8.29 (8)	8.44 (8)	8.12 (8)	8.01 (8)
Phe	4	3.58 (4)	4.49 (4)	4.74 (4)	4.21 (4)
Lys	17	10.02 (**10**)	16.93 (17)	17.35 (17)	17.2 (17)
Trp	3	ND	ND	ND	ND

^a^Amino acid sequence. Amino acid analysis of chemical modifications with ^b^AA (Lys), ^c^NPSC (Trp), ^d^NBSF (Tyr), and ^e^BPB (His). ND: nondetermined.

## Abbreviations

PLA_2_: Phospholipase A_2_
PhTX-I: *Porthidium hyoprora* myotoxin-I
EDTA: Ethylenediaminetetraacetic acid
BPB: 2,4′-Dibromoacetophenone
NBSF: 4-Nitrobenzenesulfonyl fluoride
AA: Acetic anhydride
NPSC: 2-Nitrobenzenesulfonyl chloride
CD: Circular dichroism
PBS: Phosphate-buffer saline
CK: Creatine kinase.

## References

[1] Kini RM (2003). Excitement ahead: structure, function and mechanism of snake venom phospholipase A_2_ enzymes. *Toxicon*.

[2] Pungerčar J, Križaj I (2007). Understanding the molecular mechanism underlying the presynaptic toxicity of secreted phospholipases A_2_. *Toxicon*.

[3] Gutiérrez JM, Ponce-Soto LA, Marangoni S, Lomonte B (2008). Systemic and local myotoxicity induced by snake venom group II phospholipases A_2_: comparison between crotoxin, crotoxin B and a Lys49 PLA_2_ homologue. *Toxicon*.

[4] Warrell DA (2010). Snake bite. *The Lancet*.

[5] Huang MZ, Wang QC, Liu GF (1993). Effects of an acidic phospholipase A_2_ purified from *Ophiophagus hannah* (king cobra) venom on rat heart. *Toxicon*.

[6] Atanasov VN, Danchev D, Mitewa M, Petrova S (2009). Hemolytic and anticoagulant study of the neurotoxin vipoxin and its components-basic phospholipase A_2_ and an acidic inhibitor. *Biochemistry*.

[7] Rudrammaji LMS, Machiah KD, Kantha TPK, Gowda TV (2001). Role of catalytic function in the antiplatelet activity of phospholipase A_2_ cobra (*Naja naja naja*) venom. *Molecular and Cellular Biochemistry*.

[8] Cicala C, Cirino G (1993). Phospholipase A_2_-induced hypotension in the rat and its pharmacological modulation. *General Pharmacology*.

[9] Francis BR, da Silva NJ, Seebart C, Silva LLC, Schmidt JJ, Kaiser II (1997). Toxins isolated from the venom of the Brazilian coral snake (*Micrurus frontalis frontalis*) include hemorrhagic type phospholipases A_2_ and postsynaptic neurotoxins. *Toxicon*.

[10] Ferreira T, Camargo EA, Ribela MTCP (2009). Inflammatory oedema induced by *Lachesis muta muta* (Surucucu) venom and LmTX-I in the rat paw and dorsal skin. *Toxicon*.

[11] Ponce-Soto LA, Lomonte B, Gutiérrez JM, Rodrigues-Simioni L, Novello JC, Marangoni S (2007). Structural and functional properties of BaTX, a new Lys49 phospholipase A_2_ homologue isolated from the venom of the snake *Bothrops alternatus*. *Biochimica et Biophysica Acta*.

[12] Ponce-Soto LA, Martins-de-souza D, Marangoni S (2010). Neurotoxic, myotoxic and cytolytic activities of the new basic PLA_2_ isoforms BmjeTX-I and BmjeTX-II isolated from the *Bothrops marajoensis* (marajó lancehead) snake venom. *Protein Journal*.

[13] Arni RK, Ward RJ (1996). Phospholipase A_2_—A structural review. *Toxicon*.

[14] Petan T, Križaj I, Pungerčar J (2007). Restoration of enzymatic activity in a Ser-49 phospholipase A_2_ homologue decreases its CA^2+^-independent membrane-damaging activity and increases its toxicity. *Biochemistry*.

[15] Lomonte B, Angulo Y, Sasa M, Gutiérrez JM (2009). The phospholipase A_2_ homologues of snake venoms: biological activities and their possible adaptive roles. *Protein and Peptide Letters*.

[16] Huancahuire-Vega S, Ponce-Soto LA, Martins-de-Souza D, Marangoni S (2009). Structural and functional characterization of brazilitoxins II and III (BbTX-II and -III), two myotoxins from the venom of *Bothrops brazili* snake. *Toxicon*.

[17] Jensen LB, Burgess NK, Gonda DD (2005). Mechanisms governing the level of susceptibility of erythrocyte membranes to secretory phospholipase A_2_. *Biophysical Journal*.

[18] Jenkins CM, Cedars A, Gross RW (2009). Eicosanoid signalling pathways in the heart. *Cardiovascular Research*.

[19] Cintra-Francischinelli M, Pizzo P, Angulo Y, Gutiérrez JM, Montecucco C, Lomonte B (2010). The C-terminal region of a Lys49 myotoxin mediates CA^2+^ influx in C2C12 myotubes. *Toxicon*.

[20] Huancahuire-Vega S, Ponce-Soto LA, Martins-de-Souza D, Marangoni S (2011). Biochemical and pharmacological characterization of PhTX-I a new myotoxic phospholipase A_2_ isolated from *Porthidium hyoprora* snake venom. *Comparative Biochemistry and Physiology C*.

[21] Díaz-Oreiro C, Gutiérrez JM (1997). Chemical modification of histidine and lysine residues of myotoxic phospholipases A_2_ isolated from *Bothrops asper* and B*othrops godmani* snake venoms: effects on enzymatic and pharmacological properties. *Toxicon*.

[22] Soares AM, Andrião-Escarso SH, Bortoleto RK (2001). Dissociation of enzymatic and pharmacological properties of piratoxins-I and -III, two myotoxic phospholipases A_2_ from *Bothrops pirajai* snake venom. *Archives of Biochemistry and Biophysics*.

[23] Soares AM, Giglio JR (2003). Chemical modifications of phospholipases A_2_ from snake venoms: effects on catalytic and pharmacological properties. *Toxicon*.

[24] Soons KR, Condrea E, Yang CC, Rosenberg P (1986). Effects of modification of tyrosines 3 and 62 (63) on enzymatic and toxicological properties of phospholipases A_2_ from *Naja nigricollis* and *Naja naja atra* snake venoms. *Toxicon*.

[25] Takasaki C, Sugama A, Yanagita A, Tamiya N, Rowan EG, Harvey AL (1990). Effects of chemical modifications of Pa-11, a phospholipase A_2_ from the venom of Australian king brown snake (*Pseudechis australis*), on its biological activities. *Toxicon*.

[26] Heinrikson RL, Meredith SC (1984). Amino acid analysis by reverse-phase high-performance liquid chromatography: precolumn derivatization with phenylisothiocyanate. *Analytical Biochemistry*.

[27] Schagger H, Aquila H, Von Jagow G (1987). Comassie blue-sodium dodecyl sulfate-polyacrylamide gel electrophoresis for direct visualization of polypeptides during electrophoresis. *Analytical Biochemistry*.

[28] Corrêa DHA, Ramos CHI (2009). The use of circular dichroism spectroscopy to study protein folding, form and function. *African Journal of Biochemistry Research*.

[29] Cho W, Kezdy FJ (1991). Chromogenic substrates and assay of phospholipases A_2_. *Methods in Enzymology*.

[30] Holzer M, Mackessy SP (1996). An aqueous endpoint assay of snake venom phospholipase A_2_. *Toxicon*.

[31] Borenfreund E, Borrero O (1984). In vitro cytotoxicity assays. Potential alternatives to the Draize ocular allergy test. *Cell Biology and Toxicology*.

[32] Yang CC, Chang LS (1991). Dissociation of lethal toxicity and enzymic activity of notexin from *Notechis scutatus scutatus* (Australian-tiger-snake) venom by modification of tyrosine residues. *Biochemical Journal*.

[33] Soares AM, Guerra-Sá R, Borja-Oliveira CR (2000). Structural and functional characterization of BnSP-7, a Lys49 myotoxic phospholipase A_2_ homologue from *Bothrops neuwiedi pauloensis* venom. *Archives of Biochemistry and Biophysics*.

[34] Rosenburg P, Ghassemi A, Condrea E, Dhillon D, Yang CC (1989). Do chemical modifications dissociate between the enzymatic and pharmacological activities of *β* bungarotoxin and notexin?. *Toxicon*.

[35] Andrião-Escarso SH, Soares AM, Rodrigues VM (2000). Myotoxic phospholipases A_2_ in *Bothrops* snake venoms: effect of chemical modifications on the enzymatic and pharmacological properties of bothropstoxins from *Bothrops jararacussu*. *Biochimie*.

[36] Bazaa A, Luis J, Srairi-Abid N (2009). MVL-PLA_2_, a phospholipase A_2_ from *Macrovipera lebetina transmediterranea* venom, inhibits tumor cells adhesion and migration. *Matrix Biology*.

[37] Scott DL, Achari A, Vidal JC, Sigler PB (1992). Crystallographic and biochemical studies of the (inactive) Lys-49 phospholipase A_2_ from the venom of *Agkistridon piscivorus piscivorus*. *Journal of Biological Chemistry*.

[38] Kao PH, Chen KC, Lin SR, Chang LS (2008). The structural and functional contribution of N-terminal region and His-47 on Taiwan cobra phospholipase A_2_. *Journal of Peptide Science*.

[39] Diz Filho EBS, Marangoni S, Toyama DO (2009). Enzymatic and structural characterization of new PLA_2_ isoform isolated from white venom of *Crotalus durissus ruruima*. *Toxicon*.

[40] Toyama DDO, Diz Filho EBDS, Cavada BS (2011). Umbelliferone induces changes in the structure and pharmacological activities of Bn IV, a phospholipase A_2_ isoform isolated from *Bothrops neuwiedi*. *Toxicon*.

[41] Beghini DG, Toyama MH, Hyslop S, Sodek LC, Novello, Marangoni S (2000). Enzymatic characterization of a novel phospholipase A_2_ from *Crotalus durissus cascavella* rattlesnake (Maracambóia) venom. *Journal of Protein Chemistry*.

[42] Shina R, Yates SL, Ghassemi A, Rosenberg P, Condrea E (1990). Inhibitory effect of EDTA · Ca^2+^ on the hydrolysis of synaptosomal phospholipids by phospholipase A_2_ toxins and enzymes. *Biochemical Pharmacology*.

[43] Bonfim VL, Toyama MH, Novello JC (2001). Isolation and enzymatic characterization of a basic phospholipase A_2_ from *Bothrops jararacussu* snake venom. *Protein Journal*.

[44] Yang CC, Kini RM (1997). Chemical modification and functional sites of phospholipases A_2_. *Venom Phospholipase A_2_ Enzymes: Structure, Function and Mechanism*.

[45] Rangel J, Quesada O, Gutiérrez JM, Angulo Y, Lomonte B (2011). Membrane cholesterol modulates the cytolytic mechanism of myotoxin II, a Lys49 phospholipase A_2_ homologue from the venom of *Bothrops asper*. *Cell Biochemistry and Function*.

[46] Lomonte B, Angulo Y, Calderón L (2003). An overview of lysine-49 phospholipase A_2_ myotoxins from crotalid snake venoms and their structural determinants of myotoxic action. *Toxicon*.

[47] Angulo Y, Lomonte B (2005). Differential susceptibility of C2C12 myoblasts and myotubes to group II phospholipase A_2_ myotoxins from crotalid snake venoms. *Cell Biochemistry and Function*.

[48] Gebrim LC, Marcussi S, Menaldo DL (2009). Antitumor effects of snake venom chemically modified Lys49 phospholipase A_2_-like BthTX-I and a synthetic peptide derived from its C-terminal region. *Biologicals*.

[49] Romero-Vargas FF, Ponce-Soto LA, Martins-de-Souza D, Marangoni S (2010). Biological and biochemical characterization of two new PLA_2_ isoforms Cdc-9 and Cdc-10 from *Crotalus durissus cumanensis* snake venom. *Comparative Biochemistry and Physiology C*.

[50] Costa TR, Menaldo DL, Oliveira CZ (2008). Myotoxic phospholipases A_2_ isolated from *Bothrops brazili* snake venom and synthetic peptides derived from their C-terminal region: cytotoxic effect on microorganism and tumor cells. *Peptides*.

[51] Cummings J, Hodgkinson C, Odedra R (2008). Preclinical evaluation of M30 and M65 ELISAs as biomarkers of drug induced tumor cell death and antitumor activity. *Molecular Cancer Therapeutics*.

[52] Panini SR, Yang L, Rusinol AE, Sinensky MS, Bonventre JV, Leslie CC (2001). Arachidonate metabolism and the signaling pathway of induction of apoptosis by oxidized LDL/oxysterol. *Journal of Lipid Research*.

[53] Teixeira CFP, Landucci ECT, Antunes E, Chacur M, Cury Y (2003). Inflammatory effects of snake venom myotoxic phospholipases A_2_. *Toxicon*.

[54] Teixeira C, Cury Y, Moreira V, Picolo G, Chaves F (2009). Inflammation induced by *Bothrops asper* venom. *Toxicon*.

[55] Zuliani JP, Fernandes CM, Zamuner SR, Gutiérrez JM, Teixeira CFP (2005). Inflammatory events induced by Lys-49 and Asp-49 phospholipases A_2_ isolated from *Bothrops asper* snake venom: role of catalytic activity. *Toxicon*.

[56] Vishwanath BS, Kini RM, Gowda TV (1987). Characterization of three edema-inducing phospholipase A_2_ enzymes from habu (*Trimeresurus flavoviridis*) venom and their interaction with the alkaloid aristolochic acid. *Toxicon*.

[57] Soares AM, Sestito WP, Marcussi S (2004). Alkylation of myotoxic phospholipases A_2_ in *Bothrops moojeni* venom: a promising approach to an enhanced antivenom production. *International Journal of Biochemistry and Cell Biology*.

[58] Zhao H, Tang L, Wang X, Zhou Y, Lin Z (1998). Structure of a snake venom phospholipase A_2_ modified by *p*-bromo-phenacyl-bromide. *Toxicon*.

[59] Araya C, Lomonte B (2007). Antitumor effects of cationic synthetic peptides derived from Lys49 phospholipase A_2_ homologues of snake venoms. *Cell Biology International*.

[60] Stábeli RG, Amui SF, Sant’Ana CD (2006). Bothrops moojeni myotoxin-II, a Lys49-phospholipase A_2_ homologue: an example of function versatility of snake venom proteins. *Comparative Biochemistry and Physiology C*.

